# A Research Graph dataset for connecting research data repositories using RD-Switchboard

**DOI:** 10.1038/sdata.2018.99

**Published:** 2018-05-29

**Authors:** Amir Aryani, Marta Poblet, Kathryn Unsworth, Jingbo Wang, Ben Evans, Anusuriya Devaraju, Brigitte Hausstein, Claus-Peter Klas, Benjamin Zapilko, Samuele Kaplun

**Affiliations:** 1 Australian National University, Canberra, Australia; 2 RMIT University, Melbourne, Australia; 3 Monash University, Melbourne, Australia; 4 National Computational Infrastructure, Canberra, Australia; 5 CSIRO Mineral Resources, Perth, Australia; 6 GESIS – Leibniz Institute for the Social Sciences, Köln, Germany; 7 CERN, Geneva, Switzerland

**Keywords:** Authorship, Databases, Research management

## Abstract

This paper describes the open access graph dataset that shows the connections between Dryad, CERN, ANDS and other international data repositories to publications and grants across multiple research data infrastructures. The graph dataset was created using the Research Graph data model and the Research Data Switchboard (RD-Switchboard), a collaborative project by the Research Data Alliance DDRI Working Group (DDRI WG) with the aim to discover and connect the related research datasets based on publication co-authorship or jointly funded grants. The graph dataset allows researchers to trace and follow the paths to understanding a body of work. By mapping the links between research datasets and related resources, the graph dataset improves both their discovery and visibility, while avoiding duplicate efforts in data creation. Ultimately, the linked datasets may spur novel ideas, facilitate reproducibility and re-use in new applications, stimulate combinatorial creativity, and foster collaborations across institutions.

## Background & Summary

Scientific datasets are a critical part of scientific research. Their contribution to science is as important as the journal article itself. Over the past few years there has been a widespread community effort to make datasets citable through identifiers in order to improve research transparency and facilitate science reproducibility. A recent example of such developments is the RMAP (http://rmap-project.info/rmap/) project that aims to preserve the complex relationships among traditional publications and relatively newer forms of content such as data and software^1^. Together with the availability of persistent identifiers for researchers, research articles and grants, it is now possible to promote a machine-actionable approach to address search questions such as ‘Who uses What dataset to generate What results under What funding scheme’. The pieces of the research puzzle are then joined and become traceable through linked data as long as each “W” question can be answered by a Uniform Resource Identifier (URI).

Driven by the rapid development of data storage technology, the number of research data repositories is growing rapidly. Researchers are now gaining access to a broader range of data repositories, including university data storage, discipline-specific repositories, and national (regional) level data infrastructures. The main issue is that these infrastructures often operate in silos so that their research data collections are not connected to other repositories or related research output stored in other platforms^2^.

In 2015, the Australian National Data Service (ANDS) presented the Research Link initiative as a coordinated effort to establish and maintain the connectivity between research data and other elements in the research ecosystem such as publications, grants, and researchers^3^. One of the outcomes of this initiative is the Research Data Switchboard (https://github.com/rd-switchboard) a collaborative project by ANDS and a number of other international partners in the Data Description Registry Interoperability (DDRI) Working Group of the Research Data Alliance. The working group has participants from the ANDS, Data Archiving and Networked Services (DANS - Netherlands), CERN (the European Organization for Nuclear Research), DataCite, da|ra (Germany), the Data Curation Unit of the Athena Research Centre (Greece), DataPASS, Dryad, the National Computational Infrastructure (NCI - Australia)^4^, University of Sydney (Australia), VIVO Cornell (United States), and the Clarivate Data Citation Index team. The group was established as a partnership between registries and data infrastructures after their members identified the need to link their datasets, papers, and grants to the global scholarly network.

The RD-Switchboard project addresses the issue of cross-platform discovery of research data by connecting datasets across multiple data infrastructures, very much like the "see also" section in online bookstores, where customers are invited to look at other books by the same author or within a similar or related topic. The core objective of the project is to connect different research datasets together on the basis of co-authorship or other shared properties such as a common funding source. The system aggregates links between researchers, publications, research datasets and research grants from national and international registries and utilises graph-modelling technology to identify missing links between them.

The participants in the working group have provided substantial metadata records including publications, datasets, researcher information, and grant records that are currently available in the form of an open access collection of links in the format of a graph. This graph can be used for further network analysis of research collaborations, tracing the impact of research, and finding related work across multiple repositories. In addition, this graph can be integrated with external systems using XML crosswalks, an example of such an integration is the interoperability with VIVO systems^5^ and the Scholix data-literature interlinking service^2^. One of the key benefits of this graph is that it enables discovery of related research output where there is a chain of collaborations between authors, curators, and data custodians.

## Methods

**Figure 1:**
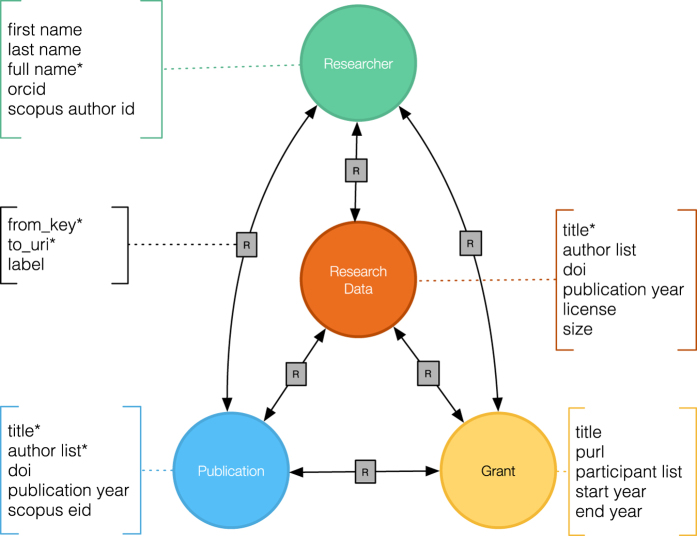
Research Graph meta model. The graph consists of connections between research datasets, publications, grants and researchers.

The graph connects research datasets to scholarly records including publications, grants, and researchers as illustrated in Fig. 1. The graph dataset described in this paper has been created using this model and the RD-Switchboard software (https://github.com/rd-switchboard). The process includes the following steps: metadata harvesting, inference, and metadata harmonisation.

### 01 Metadata harvesting

In order to build the graph we used the Open Archives Initiative Protocol for Metadata Harvesting (OAI-PMH)^6^ to harvest the metadata records from the research infrastructures. OAI-PMH is an application-independent framework to support metadata harvesting. It has been adopted by a large number of digital libraries, institutional repositories, and digital archives across the world. We used the OAI interface to harvest metadata records of publications, datasets and grants from Dryad repository, CERN INSPIRE, da|ra, and the Australian National Data Service (ANDS) repositories. The ANDS repository aggregates metadata records from more than 100 Australian research data infrastructures and Australian funding agencies. The metadata schemas associated with these sources are described in Section 0.3.

In addition, we used ORCID public JSON files igshare.com/articles/ORCID_Public_Data_File_2016/4134027) to connect researchers records to publications and datasets. Other data sources integrated into the graph are CrossRef and DataCite online services, which have been used as the authority source for retrieving metadata for digital object identifiers (DOI).

Currently, the OAI-PMH harvester of the RD-Switchboard software harvests metadata based on the XML format. However, given the rapid development of JSON-based services in the research sector, we envisage harvesting JSON-based files into the RD-Switchboard. This can be done by implementing JSON pointers (the equivalent of XPath for XML documents), which are a standardised form to identify and select a specific value inside a JSON file (http://rapidjson.org/md_doc_pointer.html). This development will be done in line with the ongoing implementation of INSPIRE^7^, the open access digital library run by a collaboration of five High Energy Physics laboratories (CERN, DESY, Fermilab, IHEP, and SLAC) that provides data mining applications and metrics for the entire corpus of literature in that area. In addition, we are working on the interoperability with JSON-LD based systems^8^. The outcome of this development can provide a standard method for exchanging records using JSON.

### 02 Inference

The RD-Switchboard inference engine obtains the connections between the nodes in the graph using three components: Node Linkings, Google Integration and Fuzzy Search. Node Linking is the main component that connects nodes based on multiple identifiers. In the case of the dataset presented in this paper, we have connected these identifiers: DOI, ORCID and PURL (Persistent URL). DOIs are typically used for publications and datasets, ORCID connects the researcher records, and PURL in our dataset identifies publicly funded grants such as http://purl.org/au-research/grants/arc/DP0774331. When two nodes have the same identifier, the inference engine creates a relationship between these nodes with relation type ‘*knownAs*’. The *knownAs* will be used by metadata harmonisation to merge publication, dataset and grant nodes.

The Google integration enables the inference engine to search for scholarly works in a subset of scholarly websites. This function creates new relationships from researchers to grants, datasets and publications. The relations created using the Google API are marked with ‘*relatedTo*’ relationships. Please note that we do not drive authorship conclusion from discovering grants or publication on the researcher profiles. The information drive from this step simply suggests that the researcher and the scholarly work are connected; however, the exact connection type is unknown.

Finally, we use a Fuzzy search algorithm to further analyse and match the title of publications and datasets with information collected about researchers using Google API. The outcome relationships from this step are also marked with *relatedTo*, and only shows potential connection rather than authoritative information about researchers’ role in the research. For further information about the function of the inference software, we recommend reviewing the code in github.com/rd-switchboard/Inference.

In summary, inference engine adds new nodes to the graph from the information available from Google API and link these nodes to existing publications, researchers and grants in the graph. Furthermore, the inference includes a process to connect existing nodes based on identifiers. These new relationships will be marked with ‘*knownAs*’ relationship type and as such can be distinguished from the authoritative links provided by repositories and publishers.

### 03 Metadata Harmonisation

In this step, we merge nodes using identifiers and aggregate the relationships to the newly formed node clusters. For the dataset presented in this paper, the nodes are combined when there is a ‘knownAs’ relationship between them, and they have DOI or ORCID. In this case, the metadata of the identifier record (e.g. Crossref DOI) would replace the individual node’s metadata. The resulting new node would inherit the relationships from the original nodes that were connected via ‘knownAs’ relations. Although this model can lead to loss of metadata from the original sources, it can enable a consistent approach to conflict resolution. For example, when a title of a publication has variation in different repositories, the title will be replaced with the metadata record from Crossref.org that is the authoritative source for most DOIs for journal articles.

One of the critical challenges in connecting scholarly works is author disambiguation, and this dataset only addresses this issue when there are ORCID records for researchers, as such, we did not merge researcher records where the ORCID identifier was missing, or it was not resolvable. This approach is a tradeoff between data efficiency and accuracy, in favour of accuracy.

One further step in metadata harmonisation is merging parallel relationships. Where there are two or more relationships between two nodes, the metadata harmonisation replaces them with a single bidirectional link with relation type *relatedTo*. The aim is to simplify the graph, hence reducing the computation cost and visualisation complexity.

The outcome of this stage is a much simpler graph where each node in the graph is tagged with one or more labels representing the information sources contributed to the content of the node. To provide consistency and enable simplicity for querying the graph we have used the Research Graph metamodel (researchgraph.org/schema/)—a light graph structure that uses CrossRef, DataCite and ORCID as the basis for representing node attributes and relationships. The code for the metadata harmonisation is part of the inference repository described in the Code Availability Section.

### Finding Connections in the Graph

The resulting graph enables finding connections between datasets using multiple degrees of separation. To demonstrate these connections, let’s define a dataset by (*D*), a publication by (*P*), a researcher by (*R*) and a grant by (*G*).

*D*_1_ − [: *citedBy*] → *P*_1_ ← [: *citedBy*] − *D*_2_: two datasets (*D*_1_ and *D*_2_) are conceptually connected because they both are cited in the same paper ( QUOTE ). This is two degrees of separation. Similar logic can be drawn from jointly funding datasets or authorship as follows

*D*_1_ − [: *fundedBy*] → *G*_1_ ← [: *fundedBy*] − *D*_2_

*D*_1_ − [: *createdBy*] → *R*_1_ ← [: *createdBy*] − *D*_2_

Note: For simplicity and improved readability, one can omit the relationship labels. Hence, the above relationship will be presented as *D*_1_ → *R*_1_ ← *D*_2_.

*D*_1_ ← *P*_1_ → *G*_1_ ← *D*_2_: two datasets (*D*_1_ and *D*_2_) are conceptually connected via a publication (*P*_1_) that cited *D*_1_ and a grant (*G*_1_) that is linked to *D*_2_ as described in *D*_2_ metadata acknowledgement. This is three degrees of separation. Similar logic can be drawn from co-authorship and connections between researchers, papers and grants. These models have been described more comprehensively in the Research Data Alliance DDRI WG recommendation^9^.

### Code availability

The RD-Switchboard source code is available under an open source license in the following GitHub repositories:

- Harvesting layer: https://github.com/rd-switchboard/Harvesters
- Inference layer: https://github.com/rd-switchboard/Inference
- Graph database interface: https://github.com/rd-switchboard/Neo4j-Browser
- Data model: https://github.com/researchgraph/schema

## Data Records

**Figure 2:**
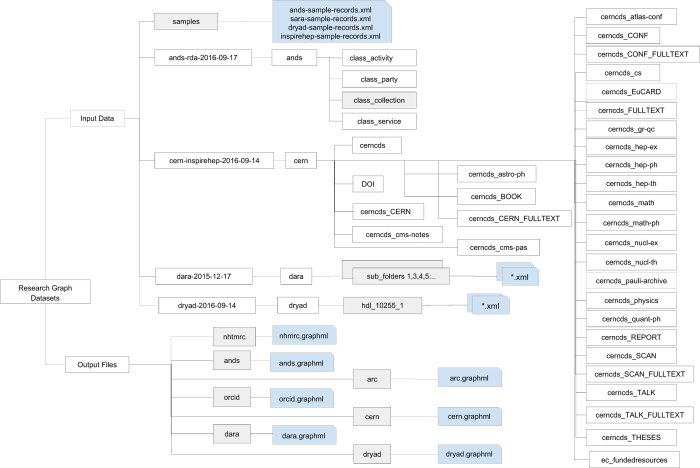
Folder structure. The schematic diagram of data files.

The datasets described in this paper are available at the figshare repository and organised into the input (Data Citation 1) and output (Data Citation 2) datasets. Figure 2 illustrates the schematic diagram of the collections. These datasets are available in a data collection accessible via figshare interface at (Data Citation 3). Please note that figshare is a DataCite member, and for this dataset, we have published the data on the figshare Monash repository.

The input datasets refer to the metadata files harvested from the repositories. They are represented following the source metadata schema and are available for download in XML format, see the ’Input’ folder in Fig. 2. They are grouped by data providers (e.g., ANDS, Dryad, and CERN). The folders organisation varies from one provider to another. In Fig. 2, data folders are indicated by grey rectangles and data files are illustrated by snip single corner rectangles. The ’sample’ folder contains an example of input XML file from all data providers. The following are metadata schemas used by the data providers:

- **Dryad**: datadryad.org/ harvested September 2016 METS format: loc.gov/METS
- **CERN**: inspirehep.net/ harvested September 2016 MARC21 format: loc.gov/MARC21/slim
- **ANDS**: researchdata.ands.org.au/ harvested September 2016 RIF-CS format: ands.org.au/online-services/rif-cs-schema
- **ARC**: arc.gov.au (RIF-CS format)/ harvested September 2016
- **NHMRC**: nhmrc.gov.au (RIF-CS format)/ harvested September 2016
- **da|ra**: da-ra.de / harvested December 2015 DARA format: da-ra.de/dara/schemadefinitions/dara.xsd

The output datasets refer to the graph files, which are available for download in GraphML -- an XML-based file format for graphs (http://graphml.graphdrawing.org). They were organized based on the data providers (Fig. 2). Each GraphML file contains nodes representing research objects (declared by the *node* element), edges connecting the nodes (declared by the *edge* element), and their attribute information (declared by a *data* element), see Listing ??. The following are attribute identifiers (denoted by the *key* attribute) associated with a data element:

- **labels**: a combination of research object types followed by the data provider.
- **title**: the title of the research object
- **original_key**: the local identifier of the research object supplied by its data provider.
- **node_type**: the research object type, e.g. grant, publication, dataset and researcher
- **node_source**: the provider of the research objec
- **relatedto**: A label that represents the relation between the source and the target research object

**Listing 1**: Example XML. Sample of a node and its edge in a directed graph.
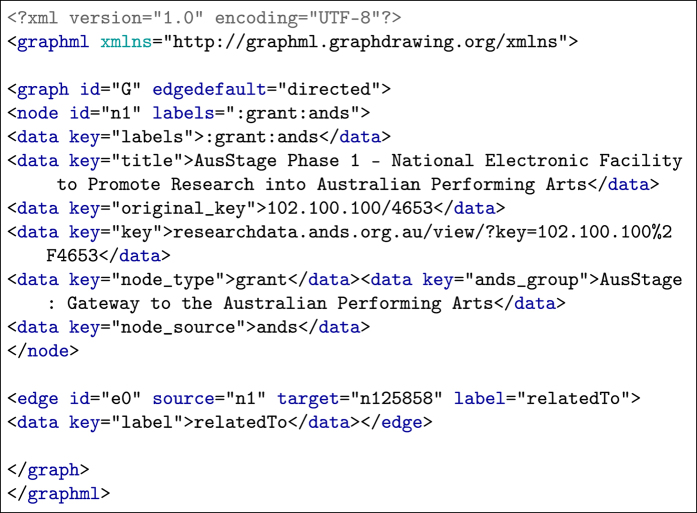


## Technical Validation

**Figure 3:**
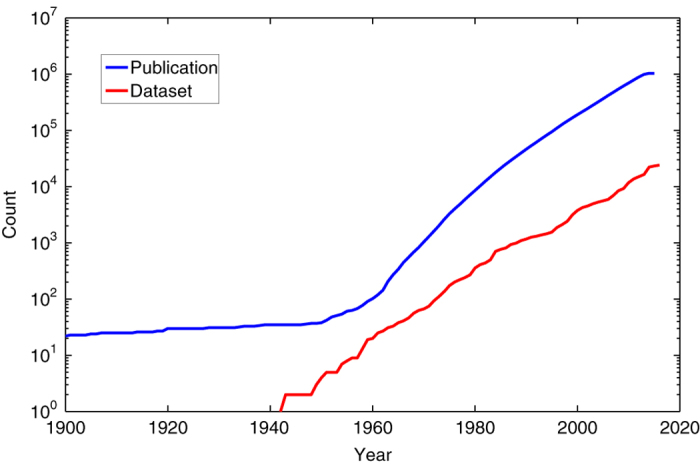
Publication trend. The logarithm of the number of publication and dataset accumulated each year.

This section provides a qualitative analysis of the graph dataset, including the number and type of connections established by RD-Switchboard. As demonstrated in Table 1, 70.6% publications have Digital Objective Identifier (DOI), while only 46% datasets have DOI. We have a high level of researcher nodes with ORCID (96.8%) identifiers, and all the provided grants are identified by PURL (Persistent URL). The high number of ORCID identifiers is mainly derived by participation of ORCID as one of the major contributors to this graph dataset. Figure 3 demonstrates the cumulative number of publications and datasets on a logarithmic scale. The published date spans from 1900 to 2016 in our database.

**Figure 4:**
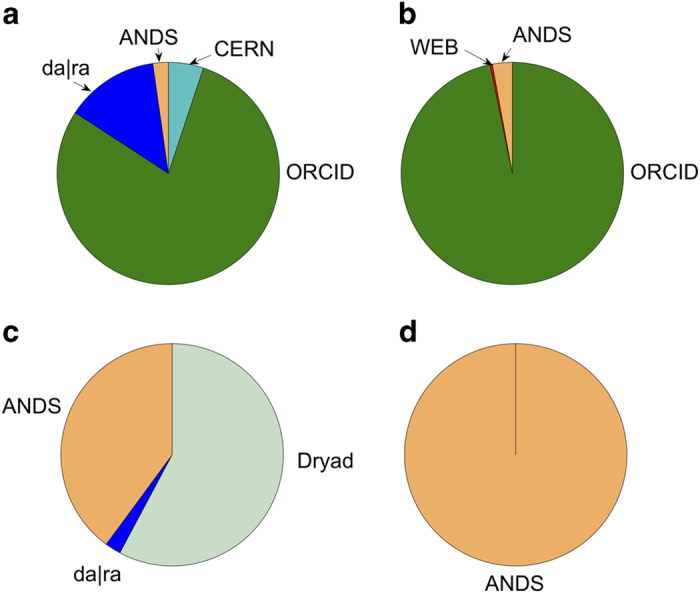
Data sources and data types. (**a**) Publication, (**b**) Researcher, (**c**) Dataset, and (**d**) Grant.

To better understand how the dataset has been formulated, Figure 4 illustrates the contribution of each source to different node types. For both publications and researchers, ORCID is the primary contributor to our graph while Da|ra, CERN, and ANDS are contributing relative smaller portions. The majority of datasets come from ANDS and Dryad. Furthermore, ANDS is the main source of grant records in our graph. Most grants in our graph come from the Australian Research Council (ARC) and National Health and Medical Research Council (NHMRC) that are mainly derived from the ANDS data source.

There are 2,797,727 relationships in total. As illustrated in Table 2, the most number of connected nodes are publications. In addition there are a large number of connections between datasets and researchers, and also researchers and grants. In contrast, there is little information available about connections between researchers and grants, and almost no connections between publication and grants.

## Usage Notes

The graph dataset includes two sets of files, the input XML and the output GraphML files. The input files are collected from different sources and come with multiple data schemas. We have worked with the input files using note++ (https://notepad-plus-plus.org) text editor mainly because of the capability for loading large size XML files. On Unix you can even use Vi editor (https://en.wikipedia.org/wiki/Vi) to open these files.

The output files are the main components of the graph dataset. These are in GraphML format and they can be opened with network visualisation tools such as gephi (https://gephi.org) and Cytoscape (https://cytoscape.org). In this section, we described how to open and analyse these files using Gephi. We use ANDS GraphML file as an example to demonstrate the process.

**Figure 5:**
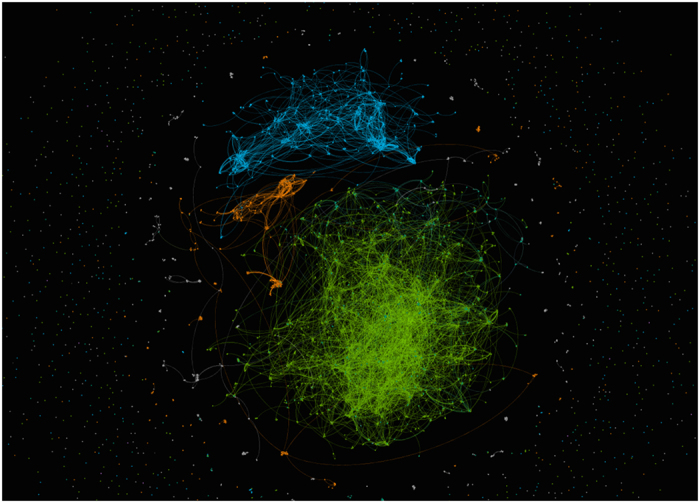
ANDS graph visualisation in Gephi. The bright blue cluster contains the grants and related records from National Health and Medical Research Council, the datasets from GeoScience Australia are highlighted by light blue, and Australian Ocean Data Network datasets are the cluster in bright orange.

- **Step 1:** Decompress the file. On Unix and Mac you can use the following command:
- unzip ands.graphml.zip
- **Step 2:** Open the file in Gephi by File and Open Menu, Navigate to the folder that you have decompressed the file, select **ands.graphml**, and click open. This will open a new Window called **Import report**.
- **Step 3:** On the **Import report** Window change the Graph Type to Undirected. The statistic should show the following:
- Number of Nodes: 211,552
- Number of Edges: 155,725
- **Step 4:** Run **Average Degree** statistic using the Statistics panel in the Overview workspace. If this panel is not visible, you can bring it to the front using Windows Menu, and submenu Statistics. The statistic result should show 1.472 Average Degree.
- **Step 5:** Remove nodes with low number of connections applying the **Degree Range** filter. You can access this filter using the Filter panel in the Overview workspace. To apply this filter, drag Degree Range filter from Topology into the Queries screen. Then click on the number QUOTE in the scale on the Degree Range Setting, and we recommend to change it to QUOTE . Then click on Filter. It might take a few seconds to complete this task. The results should be a much smaller graph (12,270 nodes and 25,109 edges) that should be manageable for most machines to visualise.
- **Step 6:** Partition nodes by their group. On the Appearance pan (available form Window menu, Appearance sub-menu), click on the Nodes tab and then on the Attribute *ands_group*.
- **Step 7:** Apply OpenOrd graph layout using the layout panel available from Window menu, Layout submenu. From the dropdown box select OpenOrd and click Run. After a couple of seconds the graph should transform to the visualisation available in Fig 5. The biggest cluster is the records from National Health and Medical Research Council that is highlighted by bright green, the two other major clusters are the datasets from GeoScience Australia (light blue) and Australian Ocean Data Network (bright orange).

Notes: The performance of Gephi very much depends on the machine CPU, the graph card and the available RAM. For this section, we have used Macbook Pro (15 inch - 2017 model) (3.1 GHz Intel Core i7) with 16GB Memory (RAM) and Radeon Pro 560 graphic card. In this section we have used Gephi version 0.9.1, the function and requirements for other version of Gephi can be different.

### Licence and Access Right

This Research Graph dataset is published under the CC-BY license, the most open and permissive of the Creative Commons licenses. The CC-BY license requires metadata aggregators to distribute the work with attribution to the original author. The CC 4.0 version now makes this requirement more flexible as attribution can be done "in any reasonable manner based on the medium, means, and context in which you share the licensed material (https://creativecommons.org/licenses/by-nc-sa/4.0/legalcode)

As regards the policies of the different registries, entities such as Dryad, da|ra or CERN (https://inspirehep.net/info/general/terms-of-use) make their metadata available under a CC0 waiver (https://creativecommons.org/about/cc0). In contrast, other entities and services such as ANDS, ARC, NHMRC or DLI have no specific policy and their metadata records come with no licenses attached. The absence of distinctive licenses for metadata records may hinder their reuse across platforms and countries and, ultimately, may trigger liability issues. To avoid legal ambiguities, a growing number of libraries and data service providers have adopted both CC0 and CC-BY, which are designed to operate globally across national jurisdictions. Nevertheless, by using them metadata aggregators also acknowledge that in many jurisdictions metadata may be subject to moral rights and copyright restrictions.

## Additional information

**How to cite this article:** Aryani A. *et al.* A Research Graph dataset for connecting research data repositories using RD-Switchboard. *Sci. Data* 5:180099 doi: 10.1038/sdata.2018.99 (2018).

**Publisher’s note:** Springer Nature remains neutral with regard to jurisdictional claims in published maps and institutional affiliations.

## Supplementary Material



## Figures and Tables

**Table 1:** Node types.

**Node Type**	**Number of Nodes**	**Number of Identifiers**
Dataset	144,354	66,371 with DOI
Publication	2,795,585	1,974,812 with DOI
Researcher	1,084,094	1,049,424 with ORCID
Grants	55,173	55,173 with PURL
Number of nodes for each type.		

**Table 2:** Connections between node types.

**Node Type**	**Dataset**	**Publication**	**Researcher**	**Grant**
Dataset	49,660	12,999	50,772	11,427
Publication	12,999	3,946	2,609,510	1
Researcher	50,772	2,609,510	213	58,804
Grant	11,427	1	58,804	397
This is an undirected graph, i.e. relations are bidirectional.				
